# Insights into the Binding of Phenyltiocarbamide (PTC) Agonist to Its Target Human TAS2R38 Bitter Receptor

**DOI:** 10.1371/journal.pone.0012394

**Published:** 2010-08-25

**Authors:** Xevi Biarnés, Alessandro Marchiori, Alejandro Giorgetti, Carmela Lanzara, Paolo Gasparini, Paolo Carloni, Stephan Born, Anne Brockhoff, Maik Behrens, Wolfgang Meyerhof

**Affiliations:** 1 Department of Molecular Genetics, German Institute of Human Nutrition Potsdam-Rebrücke (DIfE), Nuthetal, Germany; 2 Department of Biotechnology, University of Verona, Verona, Italy; 3 Statistical and Biological Physics, International School for Advanced Studies (SISSA-ISAS) and DEMOCRITOS (Modeling Center for research in atomistic Simulations), Trieste, Italy; 4 Computational Biophysics, German Research School for Simulation Sciences, Aachen, Germany; 5 Department of Reproductive and Developmental Sciences, University of Trieste, Trieste, Italy; 6 Institute for Maternal and Child Health – IRCCS “Burlo Garofolo”, Trieste, Italy; John Innes Centre, United Kingdom

## Abstract

Humans' bitter taste perception is mediated by the hTAS2R subfamily of the G protein-coupled membrane receptors (GPCRs). Structural information on these receptors is currently limited. Here we identify residues involved in the binding of phenylthiocarbamide (PTC) and in receptor activation in one of the most widely studied hTAS2Rs (hTAS2R38) by means of structural bioinformatics and molecular docking. The predictions are validated by site-directed mutagenesis experiments that involve specific residues located in the putative binding site and trans-membrane (TM) helices 6 and 7 putatively involved in receptor activation. Based on our measurements, we suggest that (i) residue N103 participates actively in PTC binding, in line with previous computational studies. (ii) W99, M100 and S259 contribute to define the size and shape of the binding cavity. (iii) W99 and M100, along with F255 and V296, play a key role for receptor activation, providing insights on bitter taste receptor activation not emerging from the previously reported computational models.

## Introduction

Humans, like other mammals, have evolutionary been prevented from the ingestion of a large variety of poisonous and toxic substances by their aversion for bitter tasting food [1]-[4]. Bitter substances bind to and are discriminated by a family of roughly ∼30 bitter taste receptors (TAS2Rs) expressed in taste receptor cells [5]–[9]. TAS2Rs belong to the super family of receptors that possess seven transmembrane helices and interact with intracellular G proteins and are therefore referred to as heptahelical or G protein-coupled receptors (GPCRs) [7], [9]. Bitter compound binding to its cognate target TAS2R initiates a downstream cascade of events inside the cell typical of GPCRs signaling pathways [10]. This cascade ultimately leads to bitter perception [2].

The knowledge of TAS2Rs' structural determinants is crucial to design rationally new chemical taste modifiers. Unfortunately, GPCRs are notoriously difficult to crystallize and so far only five independent GPCR X-ray structures have been determined. These are bovine and squid rhodopsin, turkey beta-1 and human beta-2 adrenergic receptors, and human adenosine A2 receptor [11]–[15].

Hence, insights into the molecular basis of bitter taste sensing are limited. Two studies on hTAS2R16 and hTAS2R38 relied on computations only [16], [17]. In addition, three experimentally guided structure-activity studies are available now, which all addressed hTAS2Rs distantly related to hTAS2R38 [18]–[20]. First principle [16] and homology modeling approaches based on bovine rhodopsin [17] have been used to predict the structure of the widely studied bitter taste receptor hTAS2R38 [21], [22]. Both works call upon further computational refinement and/or experimental validations. In fact, the degree of sequence conservation across the GPCRs superfamily, and the human bitter taste receptor subfamily (TAS2Rs) in particular, is very low. In this scenario, experimental validation improves greatly homology-based models [23], [24].

Here, we aim at identifying hTAS2R38 residues involved in binding to its agonist phenylthiocarbamide (PTC) as well as in receptor activation. We first use state-of-the-art bioinformatics approaches based on multiple sequence alignment across the whole family of GPCRs. However, this procedure is likely not to be sufficient to identify residues in the binding site as ligand pockets vary largely in position and orientation across this family [14]. Addressing this issue is hence aided by predicting the three-dimensional structure of the receptor, based on the former alignment and recent structural information on GPCRs along with massive virtual docking calculations. In fact, homology modeling and molecular docking has been shown to guide satisfactorily the design of site-directed mutagenesis experiments, in spite of the little power of the structural predictions [25].

The proposed receptor positions are then scrutinized by site-directed mutagenesis experiments and measurements of receptor activation by recording intracellular calcium levels following agonist administration.

## Results and Discussion

### Screening hTAS2R38 for residues involved in PTC binding

**Figure 1:**
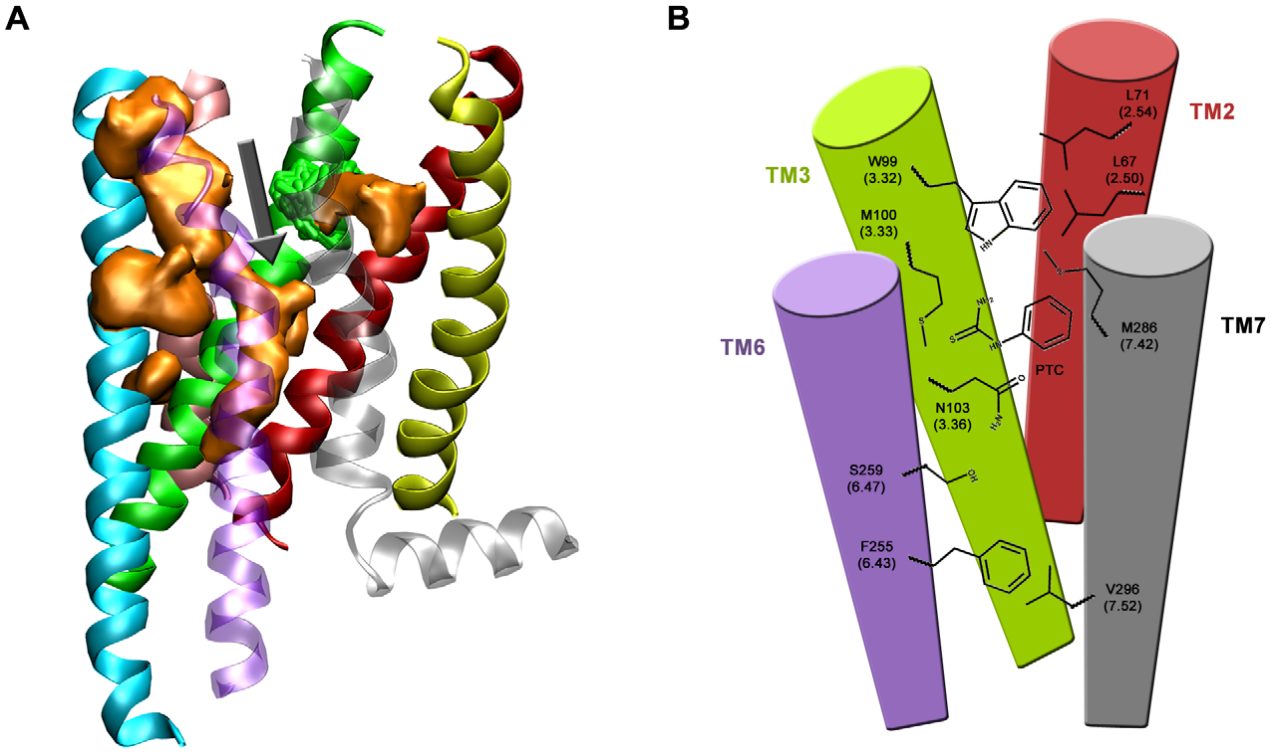
Model of the transmembrane region of hTAS2R38 predicted here. Averaged helix structures are colored as follows: TM1: lemon; TM2: red; TM3: green; TM4: pink; TM5: cyan; TM6: purple and TM7: gray. (A) The average occupancy of PTC compound during docking calculations is shown as an orange volume surface. The black arrow highlights the PTC cavity further considered in this work. (B) Schematic representation of the amino acid positions that are involved in PTC binding to hTAS2R38 receptor and activation as discussed in the text.

We constructed an ensemble of a few hundred of structural models of the receptor based on comparative homology modeling. This was achieved by aligning the hTAS2R38 sequence with those of all GPCRs whose structure has been solved (Figure S1). Several thousand reiterative virtual docking calculations followed this step in order to accommodate PTC in several putative binding sites along the entire modeled structure of the receptor (Figure S2). Of these, only one pocket is accessible from the extracellular medium for the PTC agonist (highlighted with a black arrow in Figure 1A). This cavity is located in a region equivalent to that occupied by retinal in rhodopsin [11]. The other putative binding cavities were therefore discarded. According to these predictions, PTC could bind in between TM3 and TM6 helices (Figure 1B). Residues defining the binding cavity would involve those presumably interacting with PTC: W99 (upper part of the cavity) and N103 of TM3 (positions 3.32 and 3.36 respectively, according to Ballesteros-Weinstein numbering [26]) as well as those located in the close proximity of the ligand: M100 of TM3 and S259 of TM6 (positions 3.33 and 6.47 respectively) (Figure S3).

The possible impact of specific amino acid residues in positions predicted to be critical for receptor-agonist interactions has been subsequently validated by mutagenesis experiments. By tracking intracellular calcium concentration, we established dose response curves of receptor activation following PTC administration to cells that have been previously transfected with DNA for wild-type and mutant hTAS2R38.

**Figure 2:**
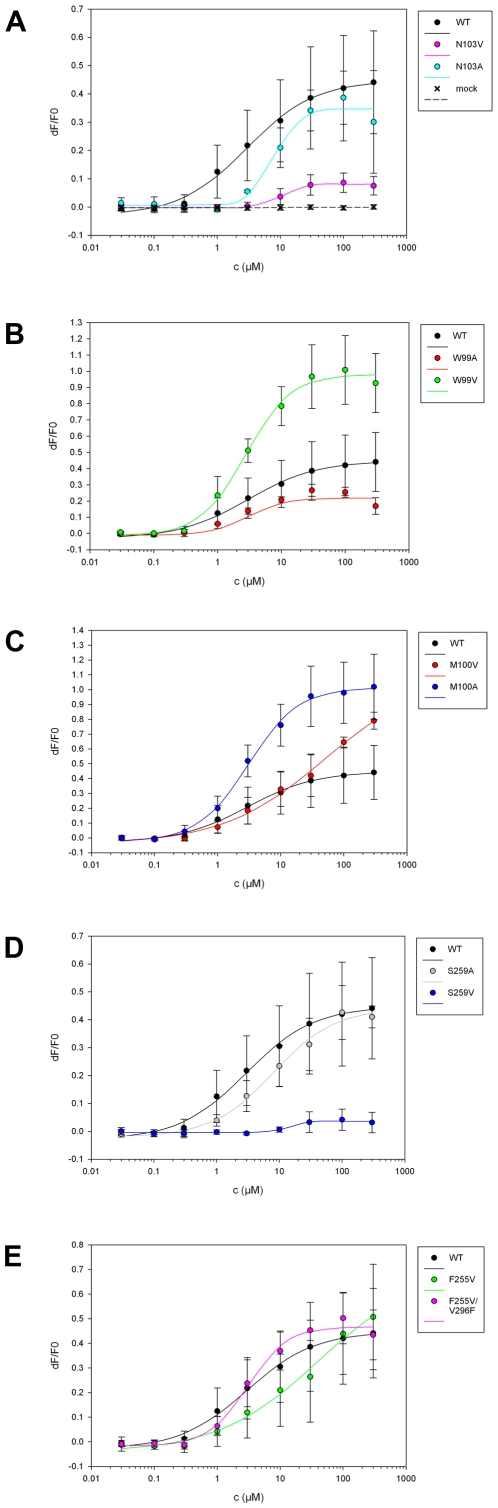
Dose-response curves of hTAS2R38 *wild type* and mutants after stimulation with increasing PTC concentrations (0 to 300 µM). Each point corresponds to the mean ± standard deviation. The mean is calculated from at least three independent experiments.

A first group of mutations were designed in order to disrupt putative interactions between the agonist and the binding pocket (Figure S3). These involve positions W99 and N103 in TM3. According to our predictions, mutations of these positions into valine or alanine would disrupt the hydrophobic interactions between PTC and the aromatic ring of W99, as well as a potential H-bond between the ligand and the amino acid side chain of N103. The collected experimental data reveal that the EC_50_ values of N103A and N103V turned out to be significantly larger than those of WT (t-test has been performed, see Table 1). Moreover, the N103V mutant was not able to recover the maximal signal amplitude of WT in the tested range of PTC concentrations (Figure 2A). These findings suggest that N103 is likely to be involved in PTC binding, consistent with our predictions. The role of N103 for binding is also corroborated by the reported models of the protein [16], [17]. On the other hand, W99V and W99A variants show a remarked increase and decrease, respectively, of the maximal signal amplitude, compared to WT, while not deviating significantly from WT in EC_50_ concentrations (Figure 2B). We hence suggest that W99 might preferentially be involved in receptor activation rather than directly in ligand binding. Also this is consistent with our predictions: W99 not only points towards the binding site, but also delimits the cavity in its upper part, being able to interact with specific residues located in the upper parts of TM2 and TM7 helices (Figure 1). When different activation states of the receptor are considered in our models, displacements longer than 3 Å are observed between W99 and helices TM2 and TM7. Thus, variations on W99 may modify the interactions between helices, altering in this way signal transduction.

**Table 1:** Amino acid positions in hTAS2R38 subjected to mutagenesis and corresponding EC_50_ values and maximum activities measured in PTC receptor activation assays.

Variant	Location	EC_50_ ( µM)	Max. Activity (dF/F0)	Expr. Rate (%)
PAV		3	0.47	31
W99A	TM3	4.25	0.25* (p = 0.019)	35
W99V	TM3	2.7	1.12* (p = 0.0006)	22
M100V	TM3	10 §	0.79	15* (p = 0.014)
M100A	TM3	3	1.01* (p = 0.0005)	15* (p = 0.012)
N103V	TM3	15* (p<0.0001)	0.09* (p = 0.0002)	17* (p = 0.006)
N103A	TM3	8* (p<0.0001)	0.38	14
F255V	TM6	4.3 §	0.5	18
F255V/V296F	TM6/TM7	2.2	0.5	13* (p = 0.0003)
S259A	TM6	5.4* (p = 0.004)	0.42	22
S259V	TM6	27* (p<0.0001)	0.04* (p = 0.0031)	10* (p = 0.031)

§ EC_50_ extrapolated.

*Statistically significant difference.

Expression rates for receptor variants (expressed in percentage). P-value is noted for statistically significant different data.

A second group of mutations target residues M100 and S259 which are located close to the putative binding cavity, but predicted not to interact directly with PTC (Figure S3). In previous reports, however, M100 has been suggested to interact with the agonist [17]. Hence, variations in these positions may help to clarify the role of these residues. The mutation of M100 into valine or alanine should change the hydrophobic interactions between the aromatic ring of PTC and M100 if present. Changing serine into valine or alanine should delete the putative H-bond between the ligand and the side chain of S259.

The EC_50_ of M100A and S259A (Figure 2C and 2D) are similar and slightly larger, respectively, than the corresponding value of WT (Table 1). This suggests that these positions would not play an active role in direct PTC-receptor interactions. On the other hand, the maximum activities of M100A and M100V are clearly higher than the activity of WT (although the elevated maximum activity observed for M100V compared to wild-type failed to reach statistical significance because higher concentrations of the agonist could not be applied), showing that this position may affect receptor activation. This can be rationalized as a steric effect in M100 position: when mutated into valine or alanine (smaller in size), wider conformational changes of the receptor are allowed.

The situation drastically changes with the S259V variant (Figure 2D). The receptor activation levels are extremely low even at high PTC concentrations (statistical analysis confirm a significant difference with respect to background activity of mock control, and the correct expression levels of the receptor are verified by immunostaining, see Table 1 and Figure S4). We suggest that ligand binding and subsequent receptor activation is very sensitive to the side chain size in this position. According to our model, S259 delimits the binding cavity in its lower part (Figure 1). Serine and alanine in the 259 position, which are similar in size, would keep the shape of the binding cavity. Instead, valine, which is larger, might disrupt severely the shape of the binding cavity. Hence, the S259V mutation is expected to impair the receptor capability to effectively bind PTC and to be activated, in an indirect fashion. Indeed, the 259 position does not participate directly to PTC-receptor interactions (see above).

In conclusion, these measurements allow us to suggest that PTC binds in between TM3 and TM6. In particular N103, belonging to TM helix 3, interacts directly with PTC. On the other hand, W99 and M100 of TM3, and S259 of TM6 are likely to be located near the binding cavity and might contribute to define its optimal shape for binding. Thus, according to our model and measurements, they do not interact directly with the ligand. All of these predictions are consistent with the available experimental data and are in line with previous suggestions [16], [17].

### Residues involved in receptor activation

**Figure 3:**
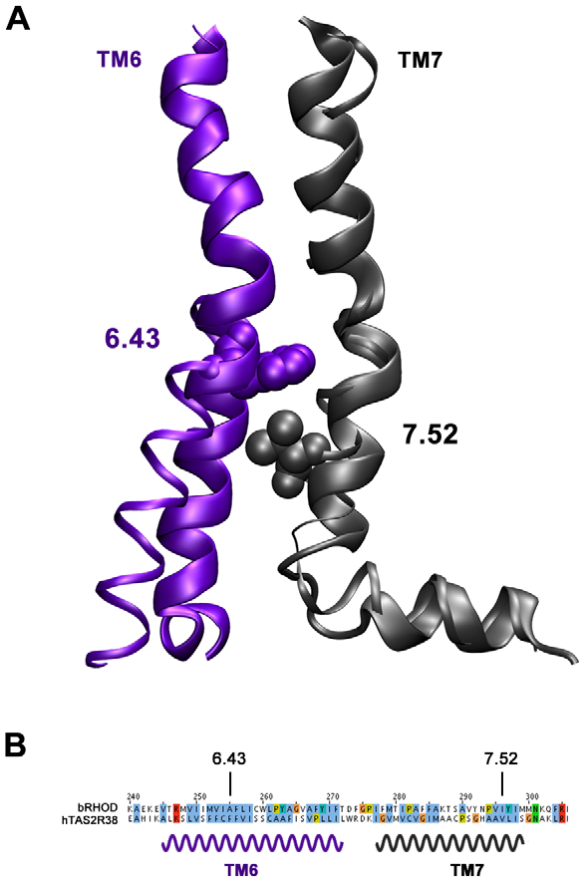
Superposition of transmembranes TM6 and TM7 in the G-protein free (thin line) and G-protein bound (thick line) states of bovine rhodopsin (extracted from PDB codes 2I37 and 3DQB respectively). (B) Multiple sequence alignment in TM6 and TM7 region (only opsin and hTAS2R38 sequences are shown).

Identification of these residues might be assisted by structural information of bovine rhodopsin [27]: The X-ray structure of this protein has been determined in two different activation states, in the presence and absence of the cognate G-protein. The comparison between the two states shows that TM5, TM6 and TM7 rearrange largely from one state to the other [27]. In particular, TM helix 6 tilts around the helical bundle upon G-protein binding (Figure 3A). The hinge point is given by residue position 6.43 according to Ballesteros-Weinstein numbering [26]. In the crystal structure of the receptor, this position faces TM helix 7 exactly at the position 7.52. According to our alignment (Figure 3B), these two positions correspond to F255 and V296, respectively, in the hTAS2R38 sequence. Thus we hypothesize that the hydrophobic interaction between F255 and V296 plays a role for hTAS2R38 receptor activation.

To test this prediction, we determined PTC dose-response curves for the mutant F255V. Replacing a rather large and aromatic residue such as phenylalanine with a significantly smaller and aliphatic residue such as valine would cause a disruption of the putative interactions between the side chains of F255 and V296. We then compared the data with those obtained for the natural hTAS2R38 variants, P49A/A262V/V296I (hTAS2R38-AVI), and A262V/V296I (hTAS2R38-PVI), which show no or reduced activity, respectively [24] (Figure S5). Although F255V variant measurements did not reach saturation, the dose-response profile of F255V roughly maintains the extrapolated EC_50_ value with respect to WT. In light of the fact that the dose-response curve does not saturate, it remains open if the maximum receptor activity is increased (Figure 2E). Extrapolating the curve, however, suggests increased signal amplitude. If this were true, F255 could indeed play an active role in receptor activation.

In order to check if F255 in TM6 actually interacts with V296 in TM7, we investigated a double cross-mutant, F255V/V296F, which is expected to recover the original interaction between both positions. The data in Figure 2E shows indeed that, consistent with our prediction, the dose-response profile of the double mutant is identical to the WT. In conclusion, the combined data from our *in silico* and *in vitro* studies suggest that the interaction between F255 and V296 may be critical for receptor activation. This result is consistent with the observation that the PVI variant (holding the V296I mutant) is less sensitive to PTC than the PAV variant [23](Figure S5).

We thus propose that hTASR38 activation upon PTC binding is reminiscent of the transition of the G-protein/opsin complex to free rhodopsin [27], with N103, directly involved in the binding, W99, M100 and S259 defining the shape of the binding cavity and, F255 and V296 participating in receptor activation (Figure 1B). Similar sequences of events also have been suggested to play a role for activation of all GPCRs [28]. Additional mutations on the putative G-protein binding region emerging from the model and in the helices involved in gating (especially helix TM7) are desirable to complement our knowledge on bitter taste receptor activation mechanism.

## Materials and Methods

### Biocomputing

All hTAS2Rs sequences were retrieved from the Uniprot [29] database using *ssearch* [30]. They were aligned with PROMALS [31]. This multiple sequence alignment was then used for the definition of the Hidden Markov profile (HMM) of hTAS2Rs. The latter was then funneled through the Hhsearch [32] program to identify the most plausible homologous structural templates. Such procedure is currently one of the best ones as evaluated from CASP7 experiment [33]. The multiple sequence alignment obtained in this way was used as the reference for the structural prediction of hTAS2R38 by homology modeling. Homology models of the receptor are here based on all the solved GPCRs structures (PDB codes 1U19, 2I37, 2RH1, 2VT4, 2ZTS, 3CAP, 3DQB, 3EML). In fact: (i) the sequence identity between target and these structural templates turned out to be as low as 13%. (ii) Some GPCRs have structural features that are distributed over different crystal structures [34]. (iii) Some GPCRs are in their activated state (rhodopsin) and others in the inactivated states (the adrenergic receptor and the adenosine receptor).

The sequence alignment between hTAS2R38 and the eight structural templates were extracted from the multiple sequence alignment considering the entire family. We then constructed 50 different conformations of hTAS2R38 (that were obtained with randomized initial structures and subsequent optimization by conjugate gradients and simulated annealing) based on each of the eight structural templates using Modeller9v3 [35]. All the three dimensional models of hTAS2R38 obtained in this way do not deviate from currently available experimental geometries (see Figure S6).

30,000 hTAS2R38/PTC adduct structures were constructed using Autodock [36]–[38]. A standard Lamarckian Genetic Algorithm was used for configurational exploration with a rapid energy evaluation using grid-based molecular affinity potentials. Electrostatic, desolvation energies and atom type affinity grid maps on the receptor were previously calculated with Autogrid [38]. We generated 100 decoys of PTC compound binding to hTAS2R38 for each of the 50 conformations. The resulting structures were then clustered according to the three dimensional localization of the ligand, regardless of the docking energies. Only the clusters in which the ligand is tightly bound in the active site cavity are discussed in this work.

### Mutant generation and transfection of cells

The 10 hTAS2R38 mutants studied here have been obtained by mutagenesis PCR using mutagenesis overlapping primers and hTAS2R38 PAV variant cDNA cloned into a pCDNA5/FRT plasmid (Invitrogen) as template. The subsequent recombinant PCR using CMV forward primer, located upstream of the cDNA sequence, and BGH reverse primer, located downstream of the cDNA sequence has been performed to fuse the overlapping mutant fragments. The mutant cDNA sequences have been digested with EcoRI and NotI restriction enzymes, to be cloned into a previously digested pCDNA5/FRT. The plasmid presented an amino terminal export tag corresponding to the first 45 amino acids of rat somatostatin receptor 3 and a carboxy terminal HSV tag [39]. The resulting mutant cDNA-constructs were sequenced to confirm their integrity. Subsequently, the 10 different mutant variants, as well as two ‘natural’ variants (AVI and PVI) have been transiently transfected with Lipofectamine2000 in HEK-293T cells stably expressing the chimeric G protein subunit Gα16gust44, very effective in coupling with bitter taste receptors [5], [40].

### Expression assay: immunocytochemistry

HEK-293T cells stably expressing the chimeric G-protein subunit Gα16gust44 have been seeded on poly-D-lysine coated coverslips and transfected with the different hTAS2R38 variants. Cells have been washed with 37°C warm PBS 24 hr after transfection and incubated on ice for 1 hr. Later, cells have been incubated on ice with biotin-labeled concanavalin A for plasma membrane staining and fixed and permeabilized with aceton-methanol 1∶1 solution. Blocking was done using 5% horse serum in PBS and antibody incubation has been performed over night at 4°C with 1∶15000 mouse anti-HSV primary antibody (Novagen). Secondary antibody incubation included both 1∶1000 Streptavidin Alexa Fluor 633 to label plasma membrane and 1∶1000 Alexa488-conjugated anti-mouse IgG (Molecular Probes) to label receptors (in 5% horse serum PBS), for 1 hr at room temperature. Coverslips have been mounted in Dako mounting medium and analyzed with a Leica confocal microscope.

### Functional Assay: Calcium imaging

Calcium imaging assay (that is based on the fluorescence emission increase of intracellular probes, Fluo4-AM dye, when bound to Ca^2+^: Because cytoplasmatic Ca^2+^ concentration increases upon GPCRs activation [41], the increase of fluorescence of the probes inside cells is associated with activation by agonist) has been performed 24 hours after transfections, three times independently for each mutant variant, using a fluorometric imaging plate reader FLIPR TETRA (Molecular Devices) and PTC as agonist in a range of 0–300 µM concentration dissolved in C1 solution. Positive (PAV variant) and negative (mock transfected) controls have been performed.

## Supporting Information

Figure S1 Multiple sequence alignment of available GPCR crystallographic structures along with human bitter taste receptor family. (See Methods for computational details).
(0.98 MB JPG)

Figure S2 Accessibility of PTC compound along different activation states of hT2R38 as modeled from different structural templates: (A) antagonist bound state. From left to right: Adenosine-receptor based model (template PDB code: 3EML), Beta-1 adrenergic receptor based model (template PDB code: 2VT4) and Beta2 adrenergic receptor based model (template PDB code: 2RH1). (B) Different activation states of rhodopsin. From left to right: Ligand free Opsin receptor based model (top) (template PDB code: 3CAP) and Ligand-free Opsin coupled to G-alpha peptide receptor based model (bottom) (template PDB code: 3DQB), Inactive Bovine (top) (template PDB code: 1U19) and Squid (bottom) (template PDB code: 2ZT3) rhodopsin receptor based model, and active-state MII Bovine rhodopsin receptor based model (template PDB code: 2I37). Averaged helix structures and residues belonging to them are colored as follows: TM1: lemon; TM2: red; TM3: green; TM4: pink; TM5: cyan; TM6: purple and TM7: gray. The average occupancy of PTC compound during docking calculations is shown as an orange volume surface.
(0.73 MB PNG)

Figure S3 Representative model of hTAS2R38 receptor bound to PTC. Putative residues important for binding and receptor activation are highlighted. The coloring scheme is as in Figure S2. Ballesteros-Weinstein numbering [25] is indicated in parenthesis.
(0.66 MB PNG)

Figure S4 Immunocytochemistry of HEK293 cells expressing the hTAS2R38 receptor PAV and mutant variants. The hTAS2R38-expressing cells are shown in green, whereas the cell surface is labeled in red.
(0.44 MB PNG)

Figure S5 Dose-response curves of hTAS2R38 variants after stimulation with increasing PTC concentrations (0 to 300 µM). Each point corresponds to the mean ± standard deviation. The mean is calculated on at least three independent experiments performed in triplicate.
(0.04 MB PNG)

Figure S6 3D structure validation of the models. All generated models have been validated against available experimental structures by means of PROCHEK server [http://www.ebi.ac.uk/thornton-srv/software/PROCHECK/]. A summary of the analysis concerning the Ramachandran angles is shown below. It indicates that our models do not deviate significantly from the usual experimental geometries (less than 2% of the amino acids).
(0.13 MB PNG)

## References

[1] Soranzo N, Bufe B, Sabeti PC, Wilson JF, Weale ME (2005). Positive selection on a high-sensitivity allele of the human bitter-taste receptor TAS2R16.. Curr Biol.

[2] Behrens M, Meyerhof W (2009). Mammalian bitter taste perception.. Results Probl Cell Differ.

[3] Meyerhof W (2005). Elucidation of mammalian bitter taste.. Rev Physiol Biochem Pharmacol.

[4] Mueller KL, Hoon MA, Erlenbach I, Chandrashekar J, Zuker CS, Ryba NJ (2005). The receptors and coding logic for bitter taste.. Nature.

[5] Behrens M, Foerster S, Staehler F, Raguse JD, Meyerhof W (2007). Gustatory expression pattern of the human TAS2R bitter receptor gene family reveals a heterogenous population of bitter responsive taste receptor cells.. J Neurosci.

[6] Shi P, Zhang J (2006). Contrasting modes of evolution between vertebrate sweet/umami receptor genes and bitter receptor genes.. Mol Biol Evol.

[7] Adler E, Hoon MA, Mueller KL, Chandrashekar J, Ryba NJ, Zuker CS (2000). A novel family of mammalian taste receptors.. Cell.

[8] Chandrashekar J, Mueller KL, Hoon MA, Adler E, Feng L (2000). T2Rs function as bitter taste receptors.. Cell.

[9] Matsunami H, Montmayeur JP, Buck LB (2000). A family of candidate taste receptors in human and mouse.. Nature.

[10] Chandrashekar J, Hoon MA, Ryba NJ, Zuker CS (2006). The receptors and cells for mammalian taste.. Nature.

[11] Palczewski K, Kumasaka T, Hori T, Behnke CA, Motoshima H (2000). Crystal structure of rhodopsin: A G protein-coupled receptor.. Science.

[12] Murakami M, Kouyama T (2008). Crystal structure of squid rhodopsin.. Nature.

[13] Rasmussen SG, Choi HJ, Rosenbaum DM, Kobilka TS, Thian FS (2007). Crystal structure of the human beta2 adrenergic G-protein-coupled receptor.. Nature.

[14] Jaakola VP, Griffith MT, Hanson MA, Cherezov V, Chien EY (2008). The 2.6 angstrom crystal structure of a human A2A adenosine receptor bound to an antagonist.. Science.

[15] Warne T, Serrano-Vega MJ, Baker JG, Moukhametzianov R, Edwards P C (2008). Structure of a beta1-adrenergic G-protein-coupled receptor.. Nature.

[16] Floriano WB, Hall S, Vaidehi N, Kim U, Drayna D, Goddard WA (2006). Modeling the human PTC bitter-taste receptor interactions with bitter tastants.. J Mol Model.

[17] Miguet L, Zhang Z, Grigorov MG (2006). Computational studies of ligand-receptor interactions in bitter taste receptors.. J Recept Signal Transduct Res.

[18] Pronin AN, Tang H, Connor J, Keung W (2004). Identification of ligands for two human bitter T2R receptors.. Chem Senses.

[19] Brockhoff A, Behrens M, Niv MY, Meyerhof W (2010). Structural requirements of bitter taste receptor activation.. Proc Natl Acad Sci U.S.A..

[20] Sakurai T, Misaka T, Ishiguro M, Masuda K, Sugawara T, Ito K, Koayashi T (2010). Characterization of the β-D-glucopyranoside binding site of the human bitter taste receptor hTAS2R16.. J Biol Chem.

[21] Kim UK, Jorgenson E, Coon H, Leppert M, Risch N, Drayna D (2003). Positional cloning of the human quantitative trait locus underlying taste sensitivity to phenylthiocarbamide.. Science.

[22] Bufe B, Breslin PA, Kuhn C, Reed DR, Tharp CD (2005). The molecular basis of individual differences in phenylthiocarbamide and propylthiouracil bitterness perception.. Curr Biol.

[23] Khafizov K, Anselmi C, Menini A, Carloni P (2007). Ligand specificity of odorant receptors.. J Mol Model.

[24] Kleinau G, Brehm M, Wiedemann U, Labudde D, Leser U, Krause G (2007). Implications for molecular mechanisms of glycoprotein hormone receptors using a new sequence-structure-function analysis resource.. Mol Endocrinol.

[25] Costanzi S (2008). On the applicability of GPCR homology models to computer-aided drug discovery: a comparison between in silico and crystal structures of the beta2-adrenergic receptor.. J Med Chem.

[26] Ballesteros JA, Weinstein H (1992). Analysis and refinement of criteria for predicting the structure and relative orientations of transmembranal helical domains.. Biophys J.

[27] Scheerer P, Park JH, Hildebrand PW, Kim YJ, Krauss N (2008). Crystal structure of opsin in its G-protein-interacting conformation.. Nature.

[28] Altenbach C, Kusnetzow AK, Ernst OP, Hofmann KP, Hubbell WL (2008). High-resolution distance mapping in rhodopsin reveals the pattern of helix movement due to activation.. Proc Natl Acad Sci U.S.A.

[29] Wu CH, Apweiler R, Bairoch A, Natale, DA, Barker WC (2006). The Universal Protein Resource (UniProt): an expanding universe of protein information.. Nucleic Acids Res.

[30] Ropelewski AJ, Nicholas HB, Deerfield DW (2004). Mathematically complete nucleotide and protein sequence searching using Ssearch.. Curr Protoc Bioinformatics.

[31] Pei J, Grishin NV (2007). PROMALS: towards accurate multiple sequence alignments of distantly related proteins.. Bioinformatics.

[32] Soding J (2005). Protein homology detection by HMM-HMM comparison.. Bioinformatics.

[33] Battey JN, Kopp J, Bordoli L, Read RJ, Clarke ND, Schwede T (2007). Automated server predictions in CASP7.. Proteins.

[34] Worth CL, Kleinau G, Krause G (2009). Comparative sequence and structural analyses of G-protein-coupled receptor crystal structures and implications for molecular models.. PLoS One.

[35] Eswar N, Webb B, Marti-Renom MA, Madhusudhan MS, Eramian D (2006). Comparative protein structure modeling using Modeller.. Curr Protoc Bioinformatics.

[36] Morris GM, Goodsell DS, Huey R, Olson AJ (1996). Distributed automated docking of flexible ligands to proteins: parallel applications of AutoDock 2.4.. J Comput Aided Mol Des.

[37] Morris GM, Huey R, Olson AJ (2008). Using AutoDock for ligand-receptor docking.. Curr Protoc Bioinformatics.

[38] Huey R, Morris GM, Olson AJ, Goodsell DS (2007). A semiempirical free energy force field with charge-based desolvation.. J Comput Chem.

[39] Bufe B, Hofmann T, Krautwurst D, Raguse JD, Meyerhof W (2002). The human TAS2R16 receptor mediates bitter taste in response to beta-glucopyranosides.. Nat Genet.

[40] Ueda T, Ugawa S, Shimada S (2005). Functional interaction between TAS2R receptors and G-protein alpha subunits expressed in taste receptor cells.. Chem Senses.

[41] Clapp TR, Stone LM, Margolskee RF, Kinnamon SC (2001). Immunocytochemical evidence for co-expression of Type III IP3 receptor with signaling components of bitter taste transduction.. BMC Neurosci.

