# Supplementary figures and images for: Insights into the Binding of Phenyltiocarbamide (PTC) Agonist to Its Target Human TAS2R38 Bitter Receptor
Source: PLoS One. 2010 Aug 25;5(8):e12394. doi: 10.1371/journal.pone.0012394 (PMC2928277; doi:10.1371/journal.pone.0012394)

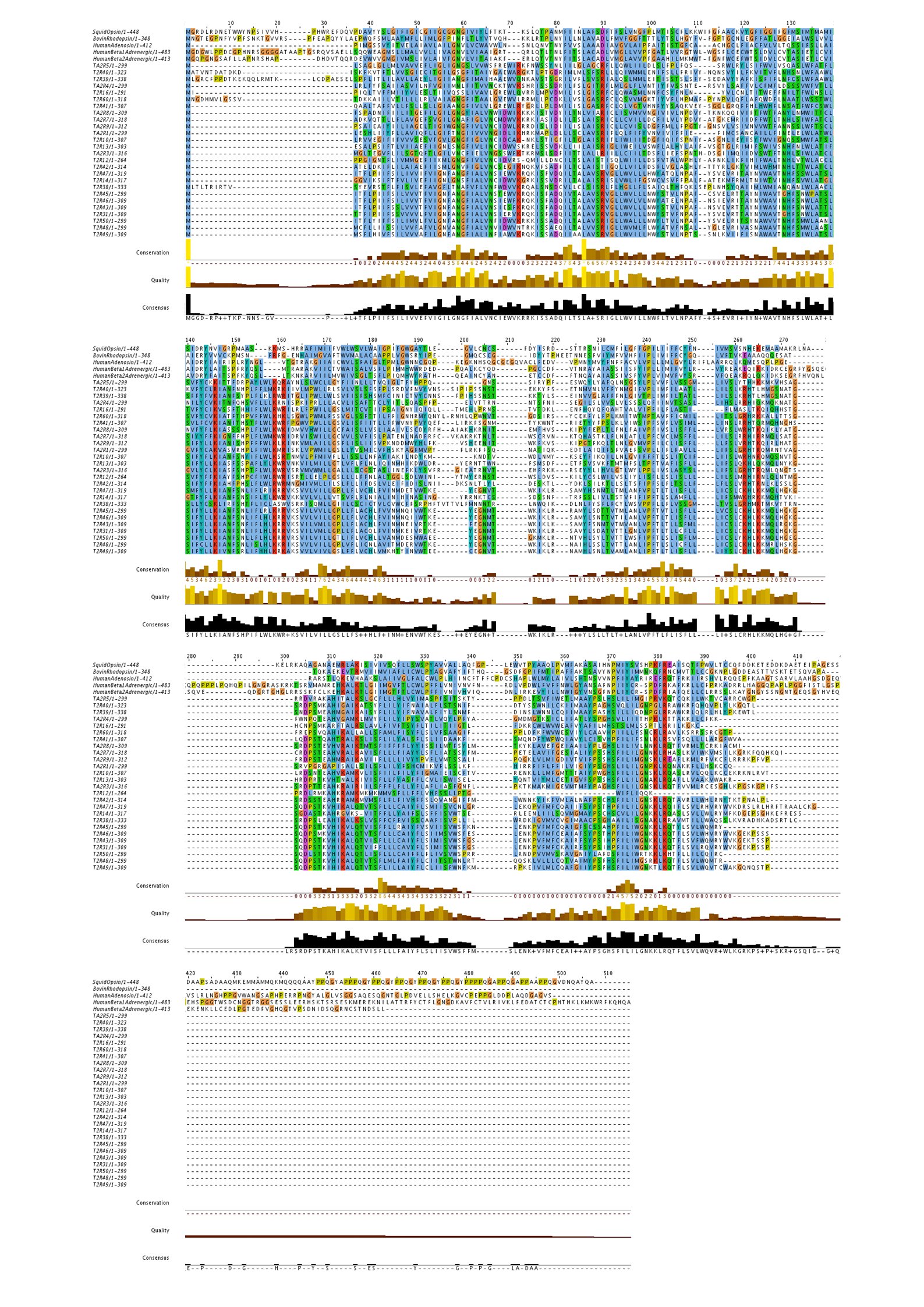

Supplement: Figure S1 — Multiple sequence alignment of available GPCR crystallographic structures along with human bitter taste receptor family. (See Methods for computational details). (0.98 MB JPG) [file pone.0012394.s001.jpg]

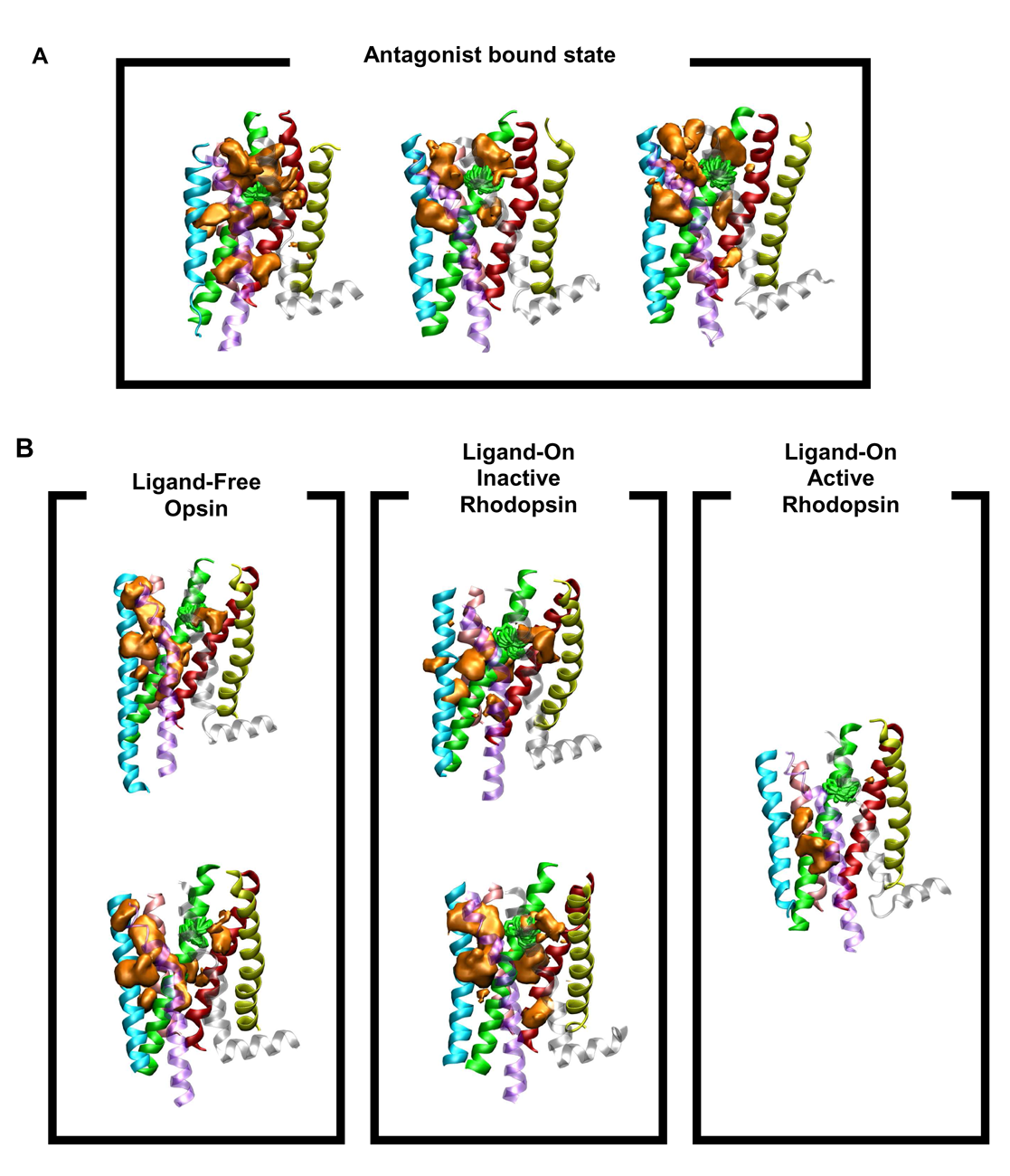

Supplement: Figure S2 — Accessibility of PTC compound along different activation states of hT2R38 as modeled from different structural templates: (A) antagonist bound state. From left to right: Adenosine-receptor based model (template PDB code: 3EML), Beta-1 adrenergic receptor based model (template PDB code: 2VT4) and Beta2 adrenergic receptor based model (template PDB code: 2RH1). (B) Different activation states of rhodopsin. From left to right: Ligand free Opsin receptor based model (top) (template PDB code: 3CAP) and Ligand-free Opsin coupled to G-alpha peptide receptor based model (bottom) (template PDB code: 3DQB), Inactive Bovine (top) (template PDB code: 1U19) and Squid (bottom) (template PDB code: 2ZT3) rhodopsin receptor based model, and active-state MII Bovine rhodopsin receptor based model (template PDB code: 2I37). Averaged helix structures and residues belonging to them are colored as follows: TM1: lemon; TM2: red; TM3: green; TM4: pink; TM5: cyan; TM6: purple and TM7: gray. The average occupancy of PTC compound during docking calculations is shown as an orange volume surface. (0.73 MB PNG) [file pone.0012394.s002.png]

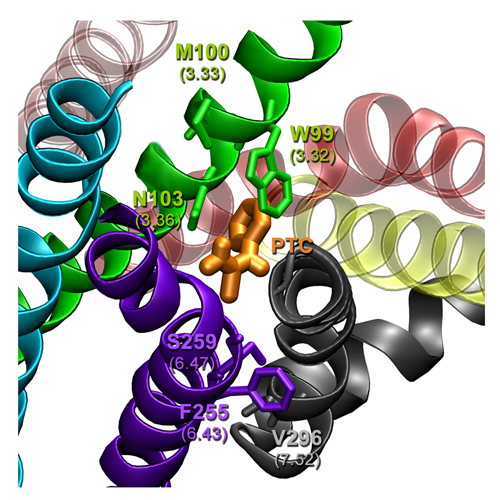

Supplement: Figure S3 — Representative model of hTAS2R38 receptor bound to PTC. Putative residues important for binding and receptor activation are highlighted. The coloring scheme is as in Figure S2. Ballesteros-Weinstein numbering [25] is indicated in parenthesis. (0.66 MB PNG) [file pone.0012394.s003.png]

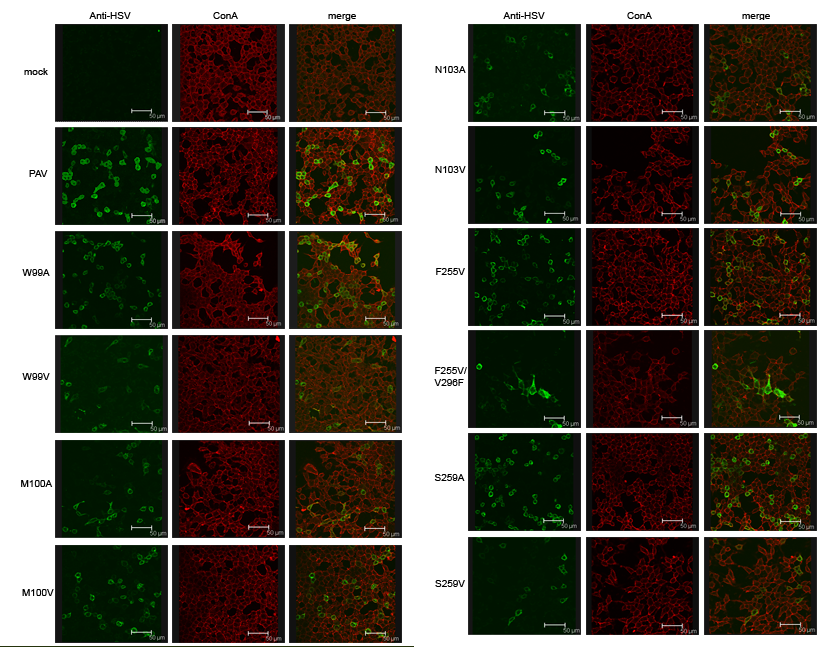

Supplement: Figure S4 — Immunocytochemistry of HEK293 cells expressing the hTAS2R38 receptor PAV and mutant variants. The hTAS2R38-expressing cells are shown in green, whereas the cell surface is labeled in red. (0.44 MB PNG) [file pone.0012394.s004.png]

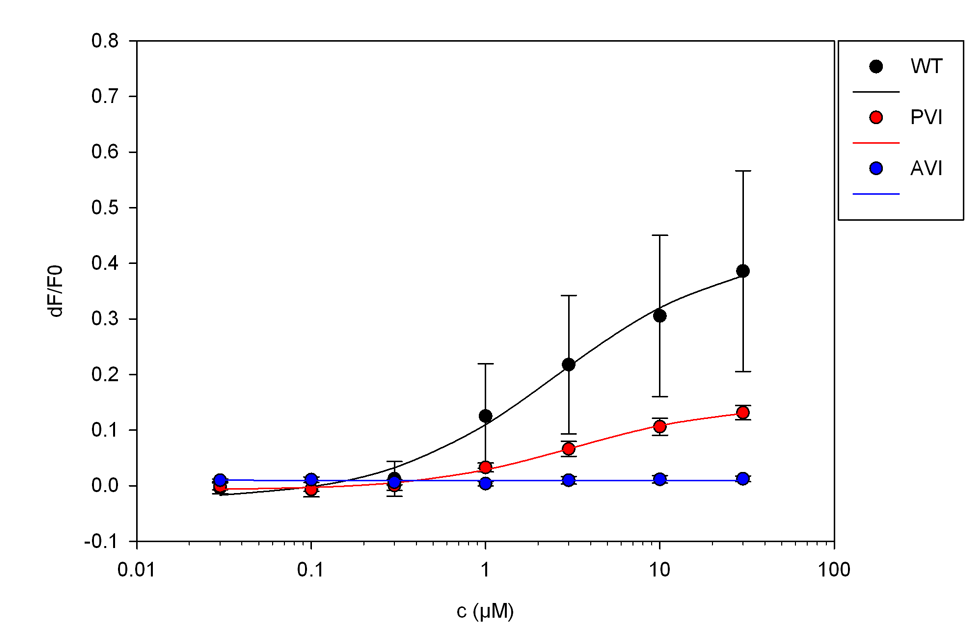

Supplement: Figure S5 — Dose-response curves of hTAS2R38 variants after stimulation with increasing PTC concentrations (0 to 300 µM). Each point corresponds to the mean ± standard deviation. The mean is calculated on at least three independent experiments performed in triplicate. (0.04 MB PNG) [file pone.0012394.s005.png]

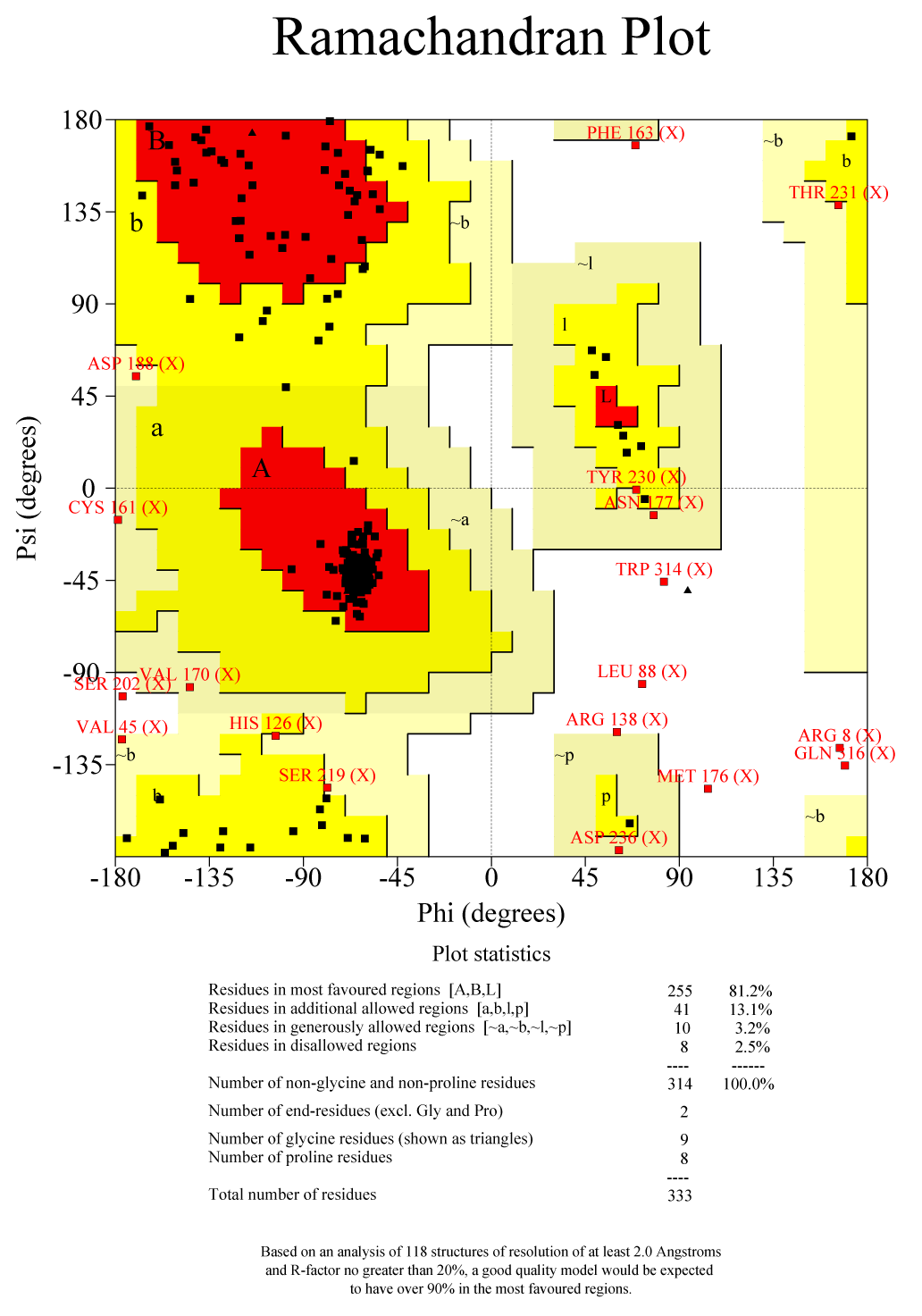

Supplement: Figure S6 — 3D structure validation of the models. All generated models have been validated against available experimental structures by means of PROCHEK server [http://www.ebi.ac.uk/thornton-srv/software/PROCHECK/]. A summary of the analysis concerning the Ramachandran angles is shown below. It indicates that our models do not deviate significantly from the usual experimental geometries (less than 2% of the amino acids). (0.13 MB PNG) [file pone.0012394.s006.png]
